# The efficacy of acupuncture in relieving postoperative pain in patients with low simple anal fistula: Protocol of a prospective, randomised, controlled trial

**DOI:** 10.1371/journal.pone.0317902

**Published:** 2025-01-24

**Authors:** Min Yang, De Zheng, Xingtao Jin, Huili Tang, Weiwei Cao, Qianqian Ye, Yin Qu, Zubing Mei

**Affiliations:** 1 Department of Anorectal Surgery, Shuguang Hospital, Shanghai University of Traditional Chinese Medicine, Shanghai, China; 2 Anorectal Disease Institute of Shuguang Hospital, Shanghai, China; Endeavour College of Natural Health, AUSTRALIA

## Abstract

**Background:**

Anal fistula surgery often leads to postoperative pain, which can hinder recovery and negatively impact patients’ quality of life. This prospective, randomised, controlled trial (RCT) aims to investigate the efficacy of acupuncture in alleviating postoperative pain and reducing the usage of analgesic medications following anal fistula surgery.

**Methods:**

This single-centre, patient-blinded, assessor-blinded, placebo-controlled randomised controlled trial (RCT) will be conducted at a tertiary referral hospital. A total of 66 patients with low simple anal fistula will be randomised at a 1:1 ratio to receive either acupuncture or sham acupuncture. The primary outcome is the difference in the numerical rating scale (NRS) pain score before and after acupuncture at 6 hours post surgery. The secondary outcomes include postoperative analgesic usage (Day 0 to Day 7), complications (including infection, urinary retention, and bleeding from Day 0 to Day 7, as well as delayed wound healing and recurrence within 3 months post surgery), sleep quality (PSQI from Day 0 to Day 7), psychological state (SDS and SAS on Day 0, Day 1, and Day 7), and overall recovery quality (QoR-15 on Day 1, Day 3, and Day 7). The statistical analysis of this trial will be conducted using SPSS software, validating the normality of data and the homogeneity of variance, and employing t-tests, Mann-Whitney U tests, chi-square tests, and repeated measures ANOVA to analyze baseline data, primary and secondary outcome indicators.

**Discussion:**

This study aims to contribute to the expanding evidence base regarding the role of acupuncture in postoperative pain management. Through a comprehensive assessment of pain relief, analgesic usage, and other recovery-related outcomes, our findings will establish a foundation for standardizing acupuncture protocols specifically tailored for anal fistula surgery patients. The strengths of this trial are rooted in its rigorous randomised controlled trial (RCT) design, comprehensive outcome measures, and focused examination of a clinically significant issue. Ultimately, the findings of this trial have the potential to offer valuable insights into the utility of acupuncture as an adjunctive therapy for postoperative pain management among anal fistula patients, thereby informing future clinical practice and research directions.

**Trial registration:**

This study was registered in the China Clinical Trial Registry on June 1, 2024, with registration number: ChiCTR2400085178.

## 1. Introduction

Anal fistula is a chronic inflammatory condition characterized by the formation of an abnormal epithelialized channel connecting the anal canal, rectum, and perianal skin. Its clinical manifestations include severe pain, discharge of secretions, faecal leakage, and incontinence [1–3]. In China, the incidence rate of anal fistula ranges from approximately 1.7% to 3.6%, with a peak occurrence observed among young and middle-aged individuals. Notably, the number of male patients is 2 to 6 times greater than that of female patients [4]. Anal fistulas rarely heal spontaneously and require timely treatment to prevent recurrence. While medication may alleviate swelling and pain during the acute phase, it is often ineffective in achieving complete resolution, thus predisposing patients to potential recurrences. Consequently, clinical practice favours surgical treatment for anal fistulas, employing techniques such as incisions and drainage, thread draining, fistulotomy, fistulectomy, and fistula tract filling, with the selection of the appropriate surgical method being contingent upon the fistula type. Postoperative complications of anal fistula surgery may include pain, bleeding, constipation, and urinary retention. A case study conducted by Nikhil C. et al. revealed that pain was the primary postoperative complication among patients under 50 years of age, significantly outweighing other common complications [5]. Therefore, the effective prevention and treatment of postoperative pain arising from anal fistulas represents an urgent matter that necessitates the attention of colorectal surgeons.

Postoperative pain following anal fistula surgery is unique owing to the distinct anatomy of the perianal region. Below the dentate line, pain sensation is governed by the autonomic nervous system, where peripheral nerves are abundant. During defecation after anal fistula excision surgery, contraction of the sphincter muscle, which stimulates the nerves and elicits pain, is necessary. If faecal residue remains on the surgical wound within the anal canal post defecation, bacteria present in the faeces can also stimulate the wounded nerves. This stimulation results in local tissue vasodilation and an increase in capillary wall permeability in the anal region [6,7]. Consequently, plasma and blood components, including neutrophils, are secreted through vessel walls into the area surrounding the anal wound [8]. Furthermore, the secretion of polypeptide substances, including bradykinin, is augmented, leading to alterations in vascular permeability, spasms of the anal smooth muscle, and vasodilation. Additionally, the increased secretion of proteinases, such as blood fibrinolytic enzymes and kinin-releasing enzymes, converts kininogen into kinin, ultimately modifying the vascular permeability and exacerbating inflammatory oedema, which contributes to pain [9]. In addition to these factors, psychological stress and surgical techniques can also influence postoperative pain [4,10].

Contemporary medical management of anal fistula postsurgical pain encompasses a diverse array of pharmacological agents and methodologies, including nonsteroidal anti-inflammatory drugs (NSAIDs), opioid analgesics, calcium channel blockers, injectable formulations, and other supplementary medications. NSAIDs, such as aspirin and indomethacin, exert their analgesic and anti-inflammatory effects by inhibiting prostaglandin synthesis, albeit with potential adverse effects on renal function [11]. Opioid analgesics, exemplified by morphine and codeine, exhibit analgesic effects on the central nervous system but may induce dependence and constipation [12,13]. Calcium channel blockers, such as diltiazem, alleviate pain by decreasing anal resting pressure [14]. Injectable agents, including compound menthol injection and compound methylene blue injection, mitigate pain through direct action on nerves or muscles [15]. Additional medications, such as gabapentin and diazepam, are also employed in pain management, each accompanied by a unique spectrum of side effects and limitations, thereby limiting their analgesic application in clinical practice [16,17].

Acupuncture has garnered widespread use as a modality for pain alleviation, and numerous studies have demonstrated the efficacy of acupuncture in mitigating postoperative pain following haemorrhoid surgeries [18,19]. The postoperative pain experienced after haemorrhoid and anal fistula surgeries is attributed primarily to the trauma inflicted upon the perianal tissues during the procedures, which subsequently elicits inflammatory responses and stimulates nerve endings. Notably, the pain mechanisms associated with these two surgeries exhibit physiological similarities. Consequently, it is reasonable to postulate that acupuncture may also be efficacious in alleviating pain following anal fistula surgery. Despite the positive outcomes reported in previously published studies [20,21], the reliability of these findings is undermined by limitations such as small sample sizes, inadequate randomization, and the absence of blinding. Owing to the inherent constraints of acupuncture treatment modalities, achieving stringent double-blind conditions in clinical practice poses a significant challenge, and there is still controversy surrounding whether its clinical efficacy can be attributed solely to the placebo effect [22,23]. Therefore, high-quality evidence is needed to confirm previous findings and develop a protocol for using acupuncture to treat postoperative pain.

For these reasons, we designed a randomised controlled trial (RCT) that incorporates rigorous randomization procedures, stringent allocation concealment, and blinding methodologies to thoroughly investigate the efficacy of acupuncture in mitigating anal fistula postsurgical pain. Our primary objectives are to ascertain whether acupuncture can serve as an effective adjunct for alleviating postoperative pain and to elucidate its significance in the recovery process following anal fistula surgery, encompassing potential improvements in postoperative sleep quality, enhancement of the psychological well-being of patients, and a reduction in the utilization of analgesic agents. This study aims to provide high-quality evidence for the standardization of acupuncture treatment in managing anal fistula postsurgical pain. Consequently, by exploring acupuncture as an adjunctive therapy for the prevention and management of anal fistula postsurgical pain, our ultimate aim is to identify safer and more efficacious treatment modalities, mitigate postoperative pain and analgesic consumption among anal fistula patients, and ultimately enhance their overall quality of life.

## 2. Methods

### 2.1 Study design

**Fig 1:**
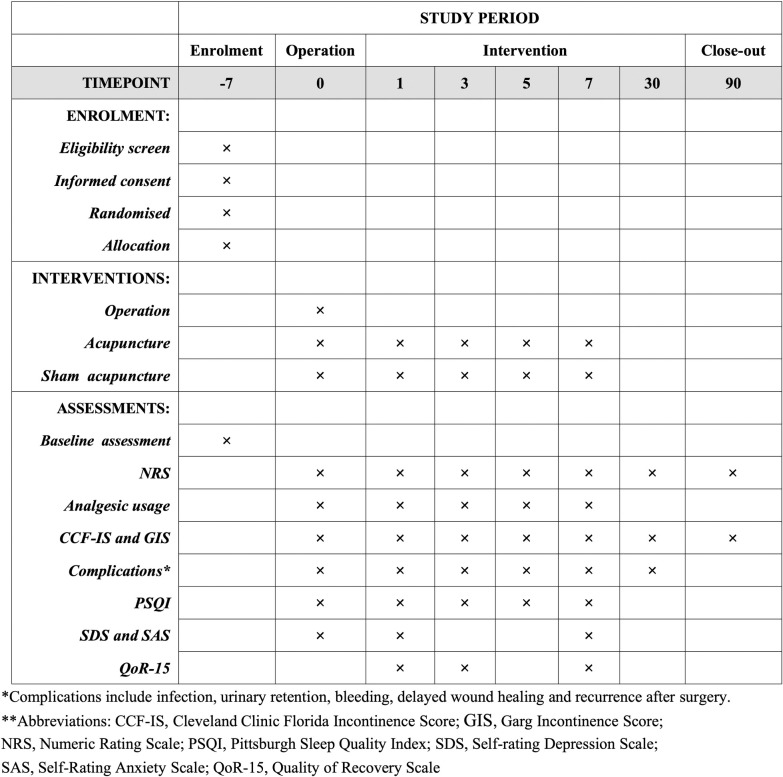
The schedule of enrolment, interventions, and assessments.

This is a single-centre, double-blinded (patient and assessor), placebo-controlled randomised controlled trial (RCT). The study will be conducted at a tertiary referral hospital. Eligible patients will be randomly assigned at a 1:1 ratio to either the acupuncture group (AP group) or the sham acupuncture group (SAP group)(see S1 and S2 Files). The design and implementation of this study will strictly adhere to the guidelines of the Helsinki Declaration [24], ensuring that all study activities comply with internationally recognized ethical standards. The enrolment period will last half a year and is expected to begin on December 1, 2024 and end on May 31, 2025. Data collection is scheduled to be completed by August 31, 2025, with preliminary conclusions expected to be drawn by September 1, 2025. The schedule of enrollment, intervention and assessment is displayed in Fig 1.

### 2.2 Sample size

The primary outcome measure is the difference in NRS score before and after analgesic intervention, assessed at 6 hours post surgery. A pilot study conducted in November 2023 at our hospital included twenty eligible patients, with ten participants allocated to each group, to investigate potential disparities in NRS scores between the acupuncture and sham acupuncture groups.

The sample size calculation was based on an analysis of data obtained from the pilot study. The analysis was conducted using PASS 2021 software (NCSS, Kaysville, Utah, USA). In this pilot study, a clinically significant treatment effect was observed, with a difference of 0.8 points in NRS scores between the two groups before and after acupuncture intervention at 6 hours post surgery. A two-sided test was used to calculate the sample size for comparing the means in a completely randomised design, with α = 0.05, β = 0.10, μ1 = 1.6, μ2 = 2.4, and σ = 0.91. By inputting these parameters into the PASS software, a sample size of N = 29 was obtained; considering a dropout rate of 10%, the final required sample size was N = 33, with 66 cases distributed equally between the two groups.

### 2.3 Enrolment

**Fig 2:**
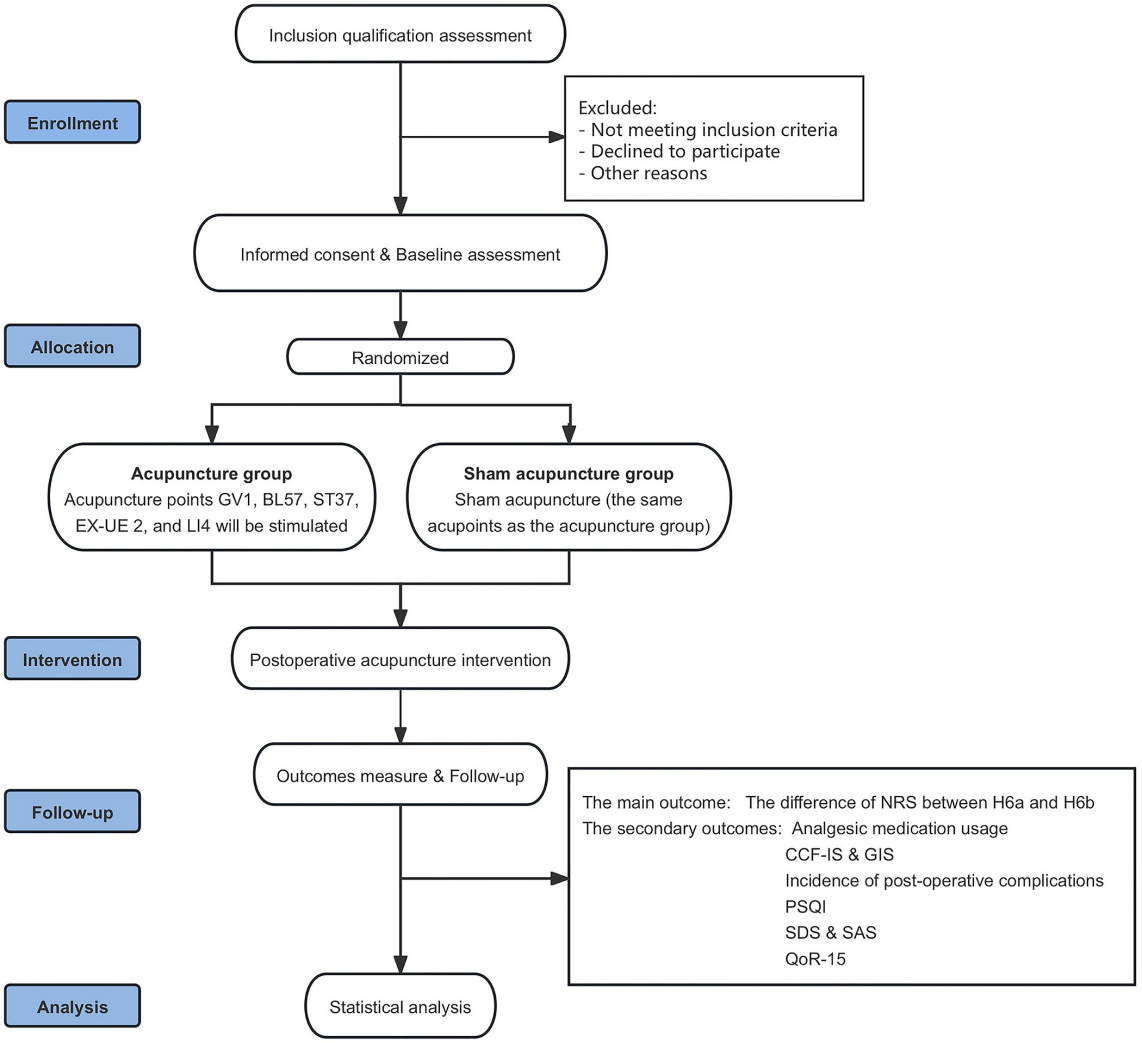
Trial flow chart.

This randomised controlled trial will be conducted at a tertiary referral hospital, where hospitalized patients who meet the eligibility criteria and are scheduled for low simple anal fistula resection will be invited to participate in the study. These patients will undergo screening by research assistants, followed by obtaining written informed consent. They will subsequently be randomly assigned to either the acupuncture group or the sham acupuncture group. The study protocol is depicted in Fig 2.

### 2.4 Inclusion and exclusion criteria

Patients eligible for inclusion must meet the following criteria:

1. Diagnostic criteria consistent with low simple anal fistula, referring to the Clinical Practice Guideline for the Management of Anorectal Abscess, Fistulas in-Ano, and Rectovaginal Fistula (2016) by the American Society of Colon & Rectal Surgeons (ASCRS) [25];
2. Distance from the external opening of the anal fistula to the anal verge of ≤3 cm;
3. Age between 18 and 65 years;
4. No absolute contraindication to anal fistulotomy;
5. Voluntary participation in the trial, ability to cooperate fully with treatment, and signed an informed consent form.
6. Postoperative NRS pain score between 4 and 9 at 6 hours after surgery.

Patients meeting any of the following criteria will be excluded from the study:

1. Patients with haemorrhoids, anal fissures, perianal abscess, anorectal tumours, Crohn’s disease, perianal skin disease, or other perianal diseases;
2. Female patients who are currently pregnant, lactating, or in a special physiological period;
3. Patients with severe hypertension, diabetes, coronary heart disease, or other chronic medical diseases, as well as those with malignant tumors, coagulation disorders, or other surgical contraindications;
4. Severe mental illness or cognitive impairment resulting in communication difficulties;
5. Patients with contraindications to acupuncture, such as ulcers, infections, or scars on the skin of the intended acupuncture site;
6. Patients who have received acupuncture treatment in the past 3 months.

### 2.5 Standard for elimination and shedding

The elimination and shedding criteria are as follows:

1. The principal investigator deems the subject unsuitable for further participation in the study, particularly in cases of poor compliance, inability to adhere to scheduled appointments, and lack of cooperation with acupuncture treatment throughout the entire duration of the study;
2. The use of other treatment methods that may result in intervention, such as the self-administration of unspecified therapeutic drugs by patients;
3. The occurrence of serious adverse events (SAEs) during the course of the study, which may be deemed unsuitable for continuation;
4. Incomplete postoperative follow-up data due to other factors;
5. Withdrawal of the patient or interruption of treatment for unknown reasons.

For all participants who drop out or discontinue the trial, we will make every effort to complete the final data collection. The reasons for dropout and the corresponding handling methods will be documented in the case report form (CRF).

### 2.6 Randomization and allocation concealment

The randomization process for this study will be conducted by an independent researcher who is not affiliated with the trial. The researcher will utilize SAS 9.3 software to generate the randomization sequence using permuted blocks with random block sizes. A total of 66 patients will be randomized in a 1:1 ratio to either the acupuncture group or the sham acupuncture group. The randomization process will ensure that each block contains an equal number of patients in both groups, with the block sizes varied randomly to prevent any predictable patterns.

To ensure allocation concealment, we will use the concealed envelope method. A total of 66 sealed, opaque envelopes will be prepared, each containing a piece of paper indicating the group assignment (A for the acupuncture group and B for the sham acupuncture group). The envelopes will be sequentially numbered according to the randomization sequence generated by SAS software. Each envelope will be opened only after the patient is enrolled and all baseline assessments are completed. The acupuncturist will be the only person aware of the group allocation when administering the treatment. After the treatment, the envelope will be resealed, and the acupuncturist will verify the allocation for each subsequent session before treatment begins. Throughout the trial, randomization will be concealed from both patients and researchers, with only the acupuncturists performing the intervention being privy to the allocation.

To achieve successful blinding, enrolled patients will be assigned to separate rooms to ensure that each patient receives acupuncture in a separate room, and a black eyeshade will be worn before acupuncture to prevent speculation and communication about trial-related information among participants or research staff.

### 2.7 Quality control

Prior to the trial, assessors and acupuncturists will undergo comprehensive training conducted by the principal investigator to ensure the trial’s quality. This training will encompass the overall flow of the trial, inclusion and exclusion criteria, precise acupoint locations, and appropriate needling depths. Acupuncture procedures will be performed in accordance with established guidelines for clinical practice [26]. The acupuncturists must possess registration credentials, hold a master’s degree, and have a minimum of 5 years of experience in acupuncture practice. Additionally, an independent researcher will handle data management, outcome measure collection, and statistical analysis.

### 2.8 Interventions

#### 2.8.1 Surgical method

Both groups of patients will undergo anal fistula excision under intravenous anaesthesia performed by the same senior and experienced surgical team. The team comprises three senior anorectal surgeons with 20 years of surgical experience, one senior interventional radiologist, one senior acupuncturist, two senior anaesthesiologists, and four experienced nurses. Once anaesthesia is deemed satisfactory, a probe will be inserted through the external opening of the fistula, passed through the fistula tract, and brought out through the internal opening. The perianal skin and subcutaneous tissue along the probe will be incised to excise the fistula tract completely.

#### 2.8.2 Postoperative basic care

After surgical treatment, all patients will receive routine anti-inflammatory therapy for two days to facilitate wound healing and expedite postoperative recovery. This intervention is aimed at mitigating the surgical-induced inflammatory response, minimizing the risk of infection, and establishing an optimal internal environment conducive to patient recovery. During the anti-inflammatory treatment period, the physician will closely monitor any changes in the patient’s condition and adjust the treatment plan accordingly. Additionally, to maintain optimal nutritional balance, the physician will recommend a light and easily digestible diet for the patient. This approach not only alleviates the gastrointestinal burden but also provides essential nutritional support.

From the first day post operation, a team of skilled nurses, assigned by the physician, will conduct regular dressing changes on the patient twice daily, in the morning and evening. During this procedure, meticulous wound cleansing will be performed by the nurse to eliminate secretions and necrotic tissue, thereby facilitating optimal wound healing.

#### 2.8.3 Postoperative pain management interventions

*1*.*Pain medication*. Patients reporting pain with NRS scores ranging from 4 to 7 will be orally administered 60 mg of loxoprofen sodium. If the NRS score exceeds 7, patients will receive an intramuscular injection of 30 mg of ketorolac tromethamine. Loxoprofen sodium is a nonsteroidal anti-inflammatory drug (NSAID) that works by inhibiting the activity of cyclooxygenase in the body, reducing the synthesis of inflammatory mediators and thereby relieving pain, inflammation, and fever [27]. Ketorolac tromethamine, a nonsteroidal anti-inflammatory drug, exerts its mechanism of action through the inhibition of cyclooxygenases (COXs), key enzymes involved in the conversion of arachidonic acid into prostaglandins, prostacyclin, and thromboxane. By effectively blocking both COX-1 and COX-2 isoforms, ketorolac tromethamine effectively alleviates pain, reduces fever, and mitigates inflammation [28,29].

*2*.*Standard acupuncture needles*. Manufactured by Suzhou Medical Supplies Factory Co., Ltd., "Hua Tuo Brand" filiform needles (size: 0.25 mm×40 mm) will be used.

**Fig 3:**
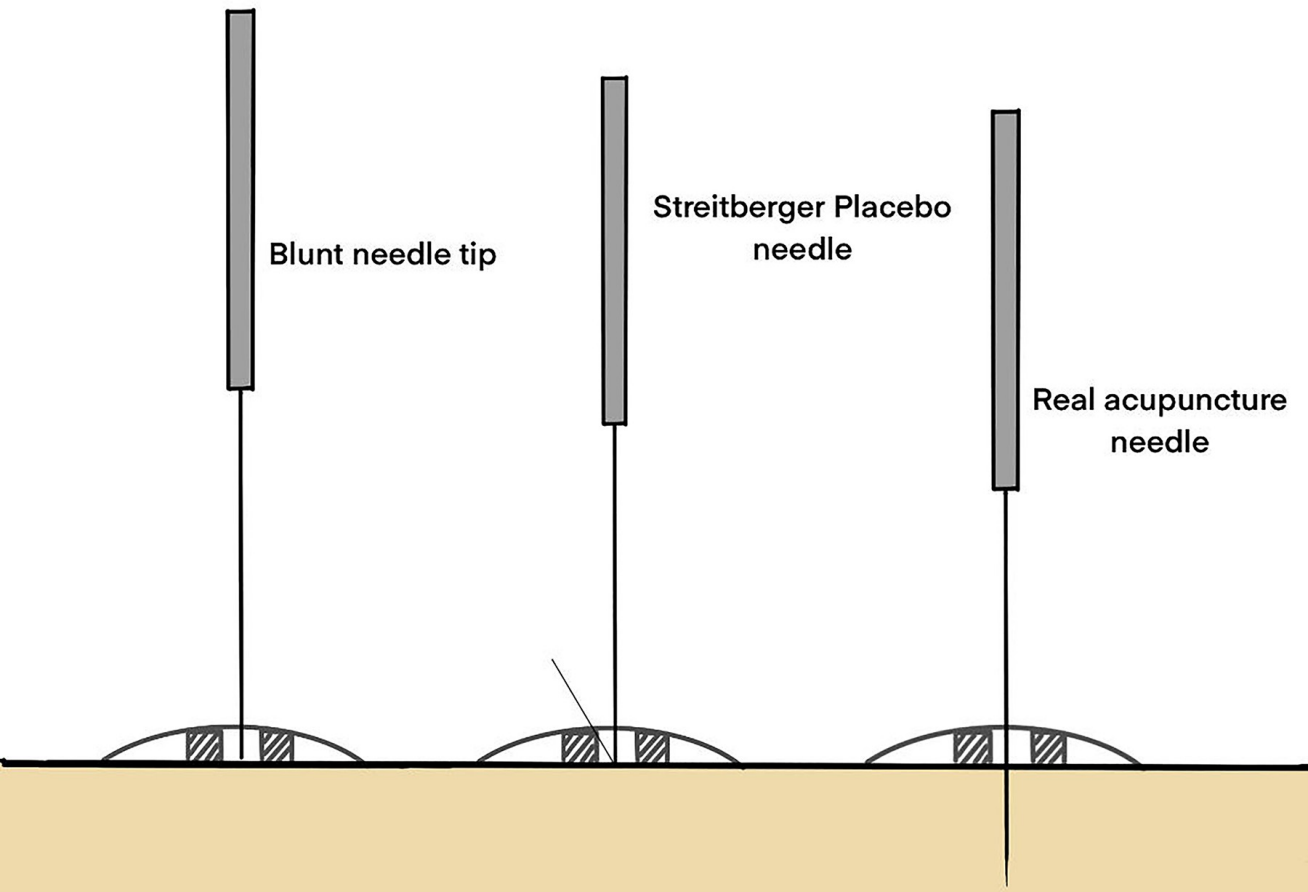
Streitberger placebo needle.

*3*.*Placebo acupuncture devices*. The Streitberger placebo needle (Streitberger placebo device [Asia-med GmbH]) resembles a genuine acupuncture needle but features a blunter tip, with the needle within the handle unsecured. Upon contact with the skin, the needle retracts into the handle and is secured with a plastic ring covered by a plastic piece. This design creates both a sensation of pricking and the illusion that the needle has penetrated the skin (see Fig 3). This placebo acupuncture device has been validated as a credible placebo control in acupuncture trials involving the Chinese population with acupuncture experience and is extensively utilized in acupuncture research [30–32].

*4*. *Selection of acupoints*. The acupoints will be identified in accordance with the method of acupoint location issued by the World Health Organization (WHO) [33]. In regard to “cun”, it is a unit of length equivalent to one-third of a decimetre [34].

1. Changqiang point (GV1)
  The Changqiang point is located on the body’s midline, specifically at the posterior end of the perineum. It can be found precisely midway between the coccyx tip and the anus. For accurate location, the midpoint between the coccyx tip and anal opening, which lies within a depression just anterior to the coccyx, should be identified.
2. Chengshan point (BL57, bilateral)
  The Chengshan point is located on the posterior aspect of the lower leg. It is situated in the depression formed below the gastrocnemius muscle when the leg is extended or the foot is flexed. To locate it accurately, we can follow these steps: identify the midpoint between the popliteal crease (the crease at the back of the knee) and the lateral malleolus (the bony prominence on the outside of the ankle). From this midpoint, palpate the area to find the most prominent bulge of the gastrocnemius muscle. Chengshan is located in the depression directly below this bulge, approximately 8 cun above the lateral malleolus.
3. Upper Juxu point (ST37, bilateral)
  The upper Juxu point is located on the anterior aspect of the lower leg. To locate it accurately, we should first identify the lateral side of the tibia (shin bone). Six cuns (approximately the width of four fingers) are measured below the lower border of the patella (kneecap). From this point, it can be palpated laterally to the anterior crest of the tibia, in the groove between the tibia and the muscle of the lower leg.
4. Erbai point (EX-UE 2, bilateral)
  The Erbai point is situated on the flexor aspect of the forearm, approximately 4 cun proximal to the palmar wrist crease. It is positioned bilaterally alongside the flexor carpi radialis tendon, with one point located on the radial side and another on the ulnar side, resulting in a total of two distinct points.
5. Hegu point (LI4, bilateral)
  The Hegu acupoint is situated on the dorsum of the hand. To accurately locate Hegu, it is essential to align the thumb and index finger, creating a subtle elevation at the apex of the muscle. The Hegu acupoint is positioned at the midpoint of the second metacarpal bone, within a depression distal to the base of both the first and second metacarpals, between the thumb and index finger. It can be found in the webbing region, precisely at its highest prominence, when bringing together the thumb and index finger.

All patients will undergo acupuncture therapy sessions, each lasting for a duration of 30 minutes, with a cumulative treatment period spanning seven days. On the designated day of surgical intervention, an acupuncture session will be administered six hours postoperatively, followed by routine acupuncture sessions conducted twice daily, once in the morning and once in the evening.

The acupuncture group will receive acupoint-specific acupuncture treatment. Patients will be positioned in a lateral decubitus position at the edge of the bed, with the knee bent and the lumbosacral region and limbs exposed. Local disinfection of the acupoint area will be performed with 75% alcohol. Sterile acupuncture needles 0.25 mm×40 mm in length will be selected to stimulate the aforementioned acupoints. Straight needling will be applied at ST 37 and EX-UE 2, with depths of 0.5–1 cun. Oblique needling will be applied at GV1, also with a depth of 0.5–1 cun. The acupoints of BL57 and LI4 will be needled straight, with a depth of 1 to 2 cun. The acupuncture protocol will adhere to the standardized permissible depth of insertion for each acupoint. Acupuncturists with over 5 years of clinical experience will perform acupuncture manipulation in a moderate manner, aiming to achieve the optimal acupuncture response known as “Deqi”, which is characterized by sensations of soreness, numbness, fullness, heaviness, and/or adjacent muscle twitching [35]. The acupuncture technique involves lifting, thrusting, and twirling the needles inserted into the acupoints for a duration of 30 minutes per session. During each session, intermittent stimulation will be applied twice every 10 minutes, with each stimulation lasting for 10 seconds.

The sham acupuncture group will receive sham acupuncture treatment. Patients in the sham acupuncture group will be positioned in the same lateral decubitus position as those in the acupuncture group, with the knee bent to expose the targeted acupoint regions. The acupoint areas will then be disinfected with 75% alcohol, mirroring the procedure for the acupuncture group. Instead of genuine acupuncture needles, Streitberger’s sham acupuncture devices will be utilized. The device will be held against the skin at the designated acupoints (GV1, BL57, ST37, EX-UE 2, and LI4), and gentle pressure will be applied to mimic the insertion process. As the tip touches the skin, the blunt needle retracts into the handle, creating the illusion of penetration without actually piercing the skin. To maintain the illusion of acupuncture, a plastic ring covered with a plastic piece will be placed over the device and fixed in place, simulating the presence of an inserted needle. This process will be repeated for each of the selected acupoints. Although no actual penetration will occur, the acupuncturist will mimic the manipulation techniques used in the acupuncture group, such as lifting, thrusting, and twirling motions, to maintain consistency with patient experience and expectations. The duration of each sham session will mirror that of the acupuncture sessions, lasting 30 minutes with intermittent "stimulation" every 10 minutes for 10 seconds each time. At the conclusion of each sham acupuncture session, the devices will be removed, and the area will be gently cleaned to remove any trace of the procedure. Patients will be informed that the treatment is complete.

### 2.9 Outcome measurements

#### 2.9.1 Assessment of baseline data

The patient’s age, sex, BMI, occupation, education background, smoking status, drinking status, disease duration, antibiotic drug use, previous perianal surgery, expectation of the analgesic effect of acupuncture, preoperative NRS pain score, sleep quality score, SAS score and SDS score will be collected and evaluated at baseline.

#### 2.9.2 Primary outcomes

The primary outcome of this study is the difference in the NRS score before (H6a) and after (H6b) acupuncture, measured at 6 hours post surgery. Compared with other measurement methods, such as the visual analogue scale, the NRS employs a numeric system that is easier for patients to grade the intensity of pain. As a patient-selected quantification of pain, the NRS score is highly feasible for both clinical research and practice. Patients can select the most appropriate number to describe their pain, and this method has been proven to be reliable, effective, and sensitive [36–38]. The NRS is an 11-point scale ranging from 0 to 10, where 0 represents no pain and 10 represents the most severe pain imaginable.

The assessment of NRS scores will be conducted at precisely defined junctures, encompassing six critical time points: preceding and subsequent to the acupuncture intervention; six hours post operation (designated H6a and H6b, respectively); and 24 hours (D1), three days (D3), five days (D5), and one week (D7) following the surgical procedure. The variance in NRS pain scores between the initial two time points serves as the primary metric, underscoring the treatment’s efficacy, whereas the comparative analysis of NRS scores at the subsequent four time points assumes the role of secondary outcome indicators. Moreover, during the patients’ hospitalization, proactive documentation of anticipatory pain intensities, particularly during defecation and dressing changes, will be meticulously maintained. Furthermore, to evaluate the long-term analgesic impact of acupuncture and ascertain its potential contribution to postoperative chronic pain, patients will undergo NRS score assessments on the 30th (D30) and 90th (D90) postoperative days, either during outpatient follow-up visits or via telephone interviews conducted by the research team.

#### 2.9.3 Secondary outcomes

*(1) Postoperative analgesic medication usage*. The use of analgesic medication (oral loxoprofen sodium or temporary use of ketorolac tromethamine muscular injection) up to the seventh day post operation will be recorded, and the usage rate of postoperative analgesics will be calculated. Analgesic medication usage rate = number of people who used additional pain medication from the first day post operation in each group/total number of people in each group.

*(2) Postoperative anal function*. The Cleveland Clinic Florida Incontinence Score (CCF-IS) and Garg Incontinence Scores (GIS) will be employed to assess anal function within 90 days post surgery. As a recently proposed method for evaluating anal function, GIS enhances the robustness of scoring while maintaining simplicity in its application, transitioning from a surgeon-centric to a patient-oriented evaluation, which represents a logical and significant advancement. Furthermore, the GIS addresses previously overlooked types of incontinence, including stress, mucus, and urge faecal incontinence, thereby providing a more comprehensive and inclusive assessment [39].

*(3) Incidence of postoperative complications*. The occurrence of postoperative complications, including infection, urinary retention, bleeding, delayed wound healing, and recurrence within one month following surgery, will also be monitored. Postoperative infection is characterized by significant pain, swelling, and the formation of pustules at the surgical site, which may be accompanied by fever. Consequently, antibiotic therapy initiation becomes necessary [40]. Postoperative urinary retention is defined as the inability to spontaneously void in the presence of bladder overdistension, necessitating catheterization for relief [41]. Postoperative haemorrhage is marked by excessive bleeding that necessitates immediate intervention, such as the application of localized pressure to achieve haemostasis at the site of bleeding [42]. Delayed wound healing is identified as failure of the wound to achieve complete re-epithelialization within 3 months post surgery. Recurrence of a fistula is identified upon its re-emergence at the previous surgical site, often necessitating diagnostic confirmation through the utilization of transrectal ultrasound (TRUS) or endorectal magnetic resonance imaging (EMRI) [43,44].

*(4) Postoperative sleep quality*. The sleep quality of both patient groups will be observed and recorded from the day prior to the surgery until the 7th postoperative night, utilizing the Pittsburgh Sleep Quality Index (PSQI). The PSQI comprises seven components, each assigned a score ranging from 0 to 3, resulting in a total PSQI score that ranges from 0 to 21 points [45]:

·Good sleep quality: (0 < PSQI ≤ 5)

·Fair sleep quality: (6 ≤ PSQI ≤ 10)

·Poor sleep quality: (11 ≤ PSQI ≤ 15)

·Very poor sleep quality: (16 ≤ PSQI ≤ 21)

*(5) Postoperative psychological state evaluation*. The Self-rating Depression Scale (SDS) [46] and the Self-Rating Anxiety Scale (SAS) [47] will be used to evaluate the patients’ psychological state during the perioperative period one day before and on Days 1 and 7 after the operation. Both scales consist of 20 items, each rated from 1 to 4 points. The sum of the scores from the 20 items is multiplied by 1.25 and then rounded to the nearest whole number to obtain the final score. Higher scores on the SAS and SDS indicate higher levels of anxiety and depression, respectively.

*(6) Evaluation of postoperative recovery quality*. The 15-item Quality of Recovery Scale (QoR-15), which assesses postoperative recovery quality across five dimensions (patient comfort, receiving help, emotional state, physical independence, and pain), shows reasonable validity and reliability [48,49]. Each item’s score ranges from 0 to 10. The QoR-15 score will be assessed at 1, 3, 7, 30 and 90 days post operation.

Upon completion of the acupuncture treatment on the seventh postoperative day, the effectiveness of the blinding procedure will be assessed by posing the following question to the patient: "During the informed consent process, you were advised that you had an equal probability of receiving acupuncture or a comparable treatment. With our study now concluded, which treatment do you believe you received?" The participants will be presented with three options: acupuncture group, sham acupuncture group, and uncertain. Furthermore, at the time of discharge, patients will be queried regarding their willingness to receive acupuncture for pain management in the future, as well as whether they would recommend acupuncture for pain control to others.

### 2.10 Follow-up schedule

To increase the quality and reliability of data collection, we will implement a standardized follow-up protocol. Patients will be explicitly informed of the necessity of attending routine follow-up appointments at two crucial postoperative time points: one month and three months after surgery. This is of paramount importance for monitoring postoperative anal pain and wound healing, facilitating the timely detection of any issues and enabling subsequent appropriate interventions.

During the follow-up process, we will prioritize core indicators, such as the patient’s NRS score for anal pain and wound healing status, while concurrently considering their overall rehabilitation progress. By inquiring about the patients’ dietary habits, defecation patterns, daily activities, and other pertinent factors, we aim to gain a comprehensive understanding of their rehabilitation trajectory and identify any potential issues. Furthermore, taking into account individual variances and specific rehabilitation requirements, we will offer personalized advice and guidance to optimize patients’ rehabilitation outcomes.

### 2.11 Safety assessments

Safety assessments, including routine blood tests, routine stool tests, biochemical examinations, and electrocardiograms, will be performed before surgery. During the trial, the occurrence of adverse reactions will be closely monitored. If symptoms such as palpitation, vomiting, fainting, skin rash, or haematoma occur, researchers should record them in detail, including the date of occurrence. In the event of adverse reactions, clinical observers can decide whether to terminate the trial on the basis of the patient’s specific conditions. If treatment is discontinued due to adverse reactions or events, the responsible researcher is tasked with conducting follow-up investigations and making detailed records.

### 2.12 Monitoring

To ensure the progress of this study, considering its clinical nature and potential intervention-related risks, we will establish an independent Data and Safety Monitoring Committee (DSMC). The committee will assume responsibility for regularly reviewing the progress of the study and closely monitoring each stage of the trial process. The members comprising the DSMC possess extensive expertise, rich experience, and a high level of professionalism in relevant fields. They will rigorously scrutinize and objectively analyse the study data. Furthermore, the DSMC will prioritize participant well-being and promptly intervene to safeguard their rights and safety should any safety concerns or adverse reactions arise during the study.

We will adhere strictly to the principles of medical ethics, ensuring the utmost respect for patient privacy and comprehensive protection of personal information for all participants. Simultaneously, we will establish a robust communication mechanism to facilitate participants’ thorough understanding of the study’s content, associated risks, and potential benefits. Moreover, participants retain the right to withdraw from the study at any time. If any problems are found or the study plan needs to be adjusted, the DSMC will have the right to decide to modify this agreement. All such modifications will undergo a rigorous review and approval process to ensure the rationality and scientific validity of the changes. Upon obtaining approval from the ethics committee, we will provide written notification to all trial participants, elucidating in detail the reasons, content, and implications of these alterations for the study.

### 2.13 Patient informed consent

Prior to engaging in the study, it is imperative that written informed consent be meticulously obtained from each patient. This consent process serves as a crucial safeguard for patients’ rights and ensures the ethical conduct of the research. During this stage, patients will be provided with comprehensive information about the study, both verbally and in written form. This information will cover the objectives of the study, the procedures involved, the potential risks and benefits, and any alternative treatment options available. The aim is to ensure that patients have a thorough understanding of all relevant details and are able to make informed decisions about their participation.

Moreover, the importance of voluntary participation will be unequivocally emphasized during the consent process. Patients will be made aware that their participation in the study is completely voluntary and that they have the right to refuse or withdraw from the trial at any time. No pressure or coercion will be exerted on patients to participate, and no reason will be required for withdrawal from the study. This ensures that patients’ autonomy and dignity are respected throughout the research process.

In addition to providing informed consent, patients will also be explicitly informed about the privacy requirements of the study. They will be made aware that their personal and medical information will be kept confidential and will only be used for the purposes specified in the consent form. Patients will also be required to provide consent for direct access to their previous medical data, which will be used to assess their eligibility for the study and to monitor any changes in their health status over the course of the trial.

### 2.14 Statistical analysis

During the study process, all data will be observed and recorded by the same individual, adhering to standardized protocols. The research data will be entered and organized by an independent third party who is not involved in this clinical trial. For statistical analysis of the collected data, we will use SPSS software (IBM SPSS 25.0, SPSS, Inc.). The normality of the measurement data will be verified using the Shapiro‒Wilk test, and the homogeneity of variance will be assessed using Levene’s test. For our primary hypothesis, we will test if acupuncture significantly reduces postoperative pain intensity at 6 hours after surgery compared to the control group using a two-sided test with a significance level of p<0.05. Our secondary hypotheses include reductions in analgesic medication usage, improvements in sleep quality, psychological well-being, overall recovery quality, reduction in postoperative complications, and improvements in anorectal function. For these hypotheses, we will adjust p-values using the Bonferroni correction to control the family-wise error rate (FWER). This adjustment will be calculated as 0.05 divided by the number of secondary hypotheses tested. The primary hypothesis is tested independently of the secondary hypotheses, and the significance threshold for the primary outcome will be unaffected by the multiple testing adjustments applied to the secondary outcomes (see S3 File).

The baseline analysis will include categorical variables such as sex, occupation, educational background, smoking habits, drinking habits, disease duration, antibiotic drug use, previous perianal surgery and expectations for the analgesic effect of acupuncture, which will be expressed as patient counts and percentages, with intergroup comparisons performed using the chi-square test. Continuous variables such as age, BMI, preoperative NRS pain scores, sleep quality scores, preoperative SAS scores, and SDS scores will be analysed on the basis of their distribution. If the data are normally distributed with homogeneity of variance, they will be reported as the mean ± standard deviation and compared using the independent samples t test; otherwise, they will be presented as medians and analysed using the Mann‒Whitney U test.

The primary outcome, the difference in the NRS score between H6a and H6b post-surgery, along with secondary outcomes (CCF-IS, GIS, PSQI, SAS, SDS, and QoR-15 scores), constitutes continuous data. These data will be described using means and standard deviations if they follow a normal distribution with equal variances, or medians and interquartile ranges otherwise. Categorical data, such as the incidence of complications and the usage rate of postoperative analgesics, will be analyzed using chi-square tests. For efficacy indicators requiring repeated measurements, such as NRS, CCF-IS, and GIS scores, we will employ both repeated-measures ANOVA and a linear mixed-effects model (LMM). The mixed-effects model will treat acupuncture therapy as a fixed effect to evaluate its overall impact across the study population. We will also include individual-level variables like age, sex, and relevant medical history as fixed effects to control for potential confounding factors, and as random effects to account for inter-patient variability. To select the most appropriate covariance structure for the LMM, we will test several structures, including unstructured, compound symmetry, and autoregressive, and choose based on the lowest Akaike Information Criterion (AIC). Bayesian Information Criterion (BIC) or likelihood ratio tests may also be utilized in cases where AIC values are closely ranked.Residual diagnostics will be performed to validate the assumptions of normality, independence, and homoscedasticity. Should any assumptions be violated, we will explore suitable data transformations or consider alternative robust statistical methods such as generalized linear mixed models (GLMM) to ensure the validity of our findings.

### 2.15 Data collection and management

To ensure accurate and efficient data management, we will implement an electronic data capture (EDC) system. The introduction of this system will not only help improve data management efficiency but also ensure data quality and security during the research process [50]. To ensure the efficient operation of the EDC system, we will employ a dedicated clinical research assistant who will be responsible for comprehensive supervision of data management and monitoring of the research progress. This individual will possess extensive experience in clinical research and demonstrate meticulous skills in data management to effectively control both data quality and research progress during the study.

The clinical research assistant will securely access the EDC system through a stringent personal electronic account, which will be safeguarded by robust confidentiality measures to ensure exclusive access and modification rights for authorized personnel. This approach guarantees the utmost security of collected patient information throughout the input and transmission process to prevent any potential data leakage or unauthorized access.

To protect the privacy and rights of patients, all data from patients participating in the study will be anonymized under the supervision of a clinical research assistant. This measure is intended to eliminate any direct association between personal information and data, thereby ensuring that the privacy of patients is fully protected. Moreover, the clinical research assistant will also be responsible for the detailed entry, comprehensive inspection and comprehensive management of the data to ensure the accuracy and completeness of the data.

### 2.16 Data availability statement

At present, our study is in the patient recruitment phase, and we have not yet obtained sufficient and meaningful data that can be shared publicly. Consequently, no research-related data have been deposited in a public repository at this time. As our trial progresses and formally commences, we will meticulously adhere to relevant requirements and deposit the minimal anonymized data set required to replicate our study findings in a stable, public repository and provide the relevant URLs, DOIs, or accession numbers. Furthermore, this study will stringently follow scientific standards and ethical guidelines to ensure the research’s quality and integrity. We will conform to CONSORT guidelines by thoroughly reporting and detailing the research design, methods, results, and conclusions.

### 2.17 Ethics statement

This study protocol was approved by the ethics committee of Shuguang Hospital affiliated with Shanghai University of Traditional Chinese Medicine (Approval Number: 2024-1519-102-01), with a validity period from May 15, 2024, to May 14, 2025. Since the ethics committee implements an annual review system, we will update the ethical approval in a timely manner.

Additionally, we will maintain an unwavering dedication to upholding the highest ethical standards throughout the entire duration of the study. We will implement comprehensive measures to protect the privacy and confidentiality of participants, ensure that informed consent is obtained transparently and fully understood by the participants, and rigorously adhere to the principles of justice and beneficence in the allocation and use of research resources. By doing so, we aim to ensure that our research is not only scientifically sound but also ethically impeccable.

### 2.18 Dissemination

The reports of the results of this study will adhere to the standards for reporting interventions in acupuncture-controlled trials and comprehensive trials and will be disseminated in a peer-reviewed journal [51,52]. Additionally, the results will be presented at suitable national and international conferences. Pertinent information regarding the trial and its outcomes will be shared with patients and disseminated through social media.

## 3. Discussion

Postoperative pain is common after anal fistula resection. It can interfere with the quality of sleep and delay postoperative recovery. If postoperative pain is not treated immediately, patients can develop long-term chronic pain. The pain caused by surgical instruments acting on the skin and muscles after anal fistula surgery shares considerable similarities with somatic and visceral pain from a physiological perspective. Upon treatment of an anal fistula with surgical instruments, the human body can activate inflammatory cells around the perianal injury, leading to the production of a large amount of inflammatory mediators, such as bradykinin, serotonin, and prostaglandins, which cause intense pain due to continuous contraction of the sphincter muscle [53]. Decomposition products of the extracellular matrix and adenosine triphosphate released by necrotic cells activate receptors on the surface of hypertrophic cells, leading to degranulation and increased release of inflammatory mediators [54]. The specific mechanism by which inflammatory factors affect postoperative pain involves stimulating nociceptors, which transmit electrical impulses to the central nervous system. Their continuous activation increases the excitability of the receptors, thus adjusting the pain threshold. Inflammatory factors increase the sensitivity of excitatory receptors, and excitatory neuropeptides such as substance P and glutamate interact with membrane receptors coupled to G proteins in neurons, lowering the action potential threshold of dorsal horn secondary neurons. This creates a "sensitization" phenomenon from normal subthreshold stimuli, leading to pain [55–57]. As anal fistulae are already associated with pain due to local inflammatory responses presurgery, this leads to increased sensitivity of surrounding nociceptors and the release of various inflammatory factors, further increasing the degree of postoperative pain [58].

We selected acupuncture as the subject of our study because previous research has demonstrated its efficacy and safety as a method of pain management. The analgesic mechanism of acupuncture in the human body involves alterations in the neuro-humoral system, affecting the regulation of the central nervous system. It enhances the synthesis, secretion, and release of cerebrospinal fluid and opioid peptides while simultaneously reducing the secretion of endogenous pain-causing substances, ultimately achieving analgesic effects [59].

GV1, an empirically validated and frequently utilized acupoint in the treatment of anorectal disorders, is located in the recess between the anus and coccyx [60]. From a local anatomical perspective, it lies proximal to branches of the anal artery and vein, extensions of the interspinous venous plexus, and the anal nerve along with posterior branches of the coccygeal nerve. Upon stimulation, the anal sphincter, which is in close proximity, promptly receives a therapeutic signal, increasing blood flow to the region [61]. Moreover, relevant research [62] has demonstrated that activating GV1 elicits parasympathetic nervous system excitation, subsequently modulating the sympathetic nervous system and regulating the concentrations of inflammatory and nociceptive mediators in the body. Consequently, this stimulation mitigates postoperative pain and facilitates bowel movements.

BL57, situated beneath the path traversed by the small saphenous vein and the posterior tibial artery and vein, holds a significant place in acupuncture therapy. The sciatic nerve, formed by the fusion of the L4–L5 and S1–S3 nerve roots, emerges from the inferior foramen of the piriformis muscle, traverses the pelvic cavity and gluteus maximus, and then bifurcates into the tibial nerve and common peroneal nerve at the popliteal fossa. The stimulation of BL57 leads to significant inhibition of spinal sensory neurons, motor neurons, and nerve ganglia within the spinal dorsal horn region [63]. Furthermore, BL57 has the ability to delay demyelination progression and promote myelin sheath regeneration following peripheral nerve injury, thereby exerting analgesic effects [64]. Fu Junwei et al. [65] innovatively employed the method of drug iontophoresis at BL57 for treatment, which not only effectively alleviated pain symptoms in patients after low anal fistula surgery but also significantly shortened the healing period.

The superficial and deep layers of the ST37 are innervated by the lateral sural cutaneous nerve, the cutaneous branches of the saphenous nerve, and the deep peroneal nerve. Stimulation of this acupoint elicits the release of the excitatory neurotransmitter substance P from central and peripheral nerve endings. This neuropeptide, which traverses various circulatory pathways, directly affects the smooth muscles of the gastrointestinal tract, inducing both "short-term" and "long-term" effects. These effects stimulate smooth muscle contraction, thereby increasing gastrointestinal motility [66]. Furthermore, manipulation of ST37 serves as a modulatory intervention in regulating colonic metabolic dysregulation through multiple metabolic pathways. This therapeutic approach significantly mitigates inflammatory mediator levels, underscoring its potential in ameliorating inflammatory states within the colon [67].

The superficial layer of the EX-UE2 region is innervated by the cutaneous branches of the ulnar and radial nerves of the forearm, whereas its deep layer harbours the radial artery trunk, superficial radial nerve, and median nerve, accompanied by muscular branches of the median nerve and the anterior interosseous artery. In the traditional Chinese medical literature, this acupoint holds prominence in the treatment of anorectal disorders [68]. Contemporary research has further substantiated that acupuncture at EX-UE 2 notably alleviates postoperative pain, haemorrhage, and oedema associated with anorectal diseases, underscoring its therapeutic efficacy in managing such conditions [69,70].

LI4, a pivotal component in the armamentarium of acupuncture analgesia, resides adjacent to the second metacarpal bone. Upon insertion of the acupuncture needle into this site, it elicits stimulation of the periosteum, thereby precipitating an intricate cascade of biochemical reactions that facilitate the release of vasoactive substances. These physiological sequelae culminate in a reduction in pain-mediating factors such as bradykinin and serotonin, thereby achieving the desired analgesic effect [71]. Research conducted by Hou Jinwen et al. [72] has illuminated the profound implications of stimulating the Hegu acupoint through acupuncture. These findings suggest that such stimulation activates crucial neural networks that are integral to the modulation of nociceptive impulse transmission. This activation not only instigates a transient alleviation of chronic pain but also observably attenuates signals emanating from the anterior cingulate cortex and the visual cortex, thereby attesting to this acupoint’s marked sedative properties.

This study has several strengths. Notably, this is the first study to combine acupuncture therapy with postoperative analgesic intervention following excision of low simple anal fistulas and the first randomised controlled trial to assess its safety and efficacy. As such, this study aims to contribute high-quality, peer-reviewed evidence to guide clinical practice, addressing a critical gap in the existing medical literature. Furthermore, the trial incorporates comprehensive outcome measures that evaluate both subjective and objective aspects. By employing this multifaceted approach to outcome evaluation, this study ensures a more holistic understanding of the efficacy of acupuncture therapy for these patients.

However, there are several limitations to this study. As a pilot trial with a limited sample size designed to gather preliminary data, the study’s capacity for controlling potential confounding factors is somewhat restricted. Although we will incorporate demographic and clinical characteristics such as age, sex, and BMI into our baseline analysis, the scope of our study does not extend to a comprehensive examination of all possible confounders. However, our study’s randomised controlled trial design, in which participants are randomly assigned to either the intervention or control group, ensures an equal distribution of both known and unknown confounding variables across the groups. This randomization process is crucial for maintaining the internal validity of the study, enabling us to attribute differences in outcomes more confidently to the intervention itself rather than to external variables. Follow-up studies can expand the sample size, expand the scale of clinical trials, and improve the accuracy of the test results. In addition, this trial does not consider some factors that may affect pain after anal fistula resection, such as the dose of anaesthetic drugs, the location and depth of the incision, or differences in patient physique. Moreover, the acupoints used in this trial are limited, and subsequent research can explore whether more acupoints can be used to relieve sphincter spasm and affect postoperative anal function. Additionally, the postoperative follow-up period is limited to three months, lacking any extended long-term observational data. The study’s design, being a single centre, also hampers the broad application and generalizability of the results. Thus, further verification through multicentre and large-sample clinical trials is necessary. Finally, in the current experimental design, the observation of pain relies primarily on subjective pain rating systems, which offer a direct reflection of patients’ pain perception but may have limitations in terms of comprehensiveness and objectivity. Subjective ratings are susceptible to interference from patients’ emotions, comprehension abilities, and expressive capacities, rendering it challenging to directly capture the physiological alterations and neural mechanisms underlying pain. To facilitate a more comprehensive and scientific assessment of pain, future trials should incorporate multiple objective indicators as complementary measures. Specifically, this can be achieved by monitoring physiological markers such as heart rate variability and blood pressure fluctuations to objectively quantify pain intensity. Additionally, behavioural assessments, including pain avoidance behaviour tests and pain threshold determinations, can indirectly reflect the actual pain experience. Furthermore, monitoring changes in inflammatory factors in the blood can elucidate the relationship between the body’s inflammatory state and pain. By integrating subjective ratings with a diverse array of objective indicators, a multimodal pain assessment framework can be established. This framework, through comprehensive analysis of data sourced from multiple avenues, aims to unveil the essence and characteristics of patients’ pain in a more holistic manner, thereby enhancing the accuracy and reliability of pain assessment.

To the best of our knowledge, few randomised controlled trials have specifically evaluated the efficacy of acupuncture versus sham acupuncture in managing postoperative pain following anal fistula surgery. Our study aims to supplement and enrich the literature by conducting a rigorous trial that not only focuses on the evolution of postoperative pain but also delves into the quality of sleep, patients’ psychological well-being, and postoperative anal function. By comprehensively assessing these outcomes, we anticipate that our findings will provide valuable evidence that can inform the use of acupuncture as a potential adjuvant therapy for postoperative pain management, with the ultimate goal of minimizing the need for postoperative analgesic drugs while simultaneously alleviating patients’ pain and enhancing their overall recovery quality.

## Supporting information

S1 File SPIRIT checklist. (DOCX)

S2 File Study protocol approved by the ethics committee. (PDF)

S3 File Statistical analysis plan. (DOCX)

## Abbreviations

NRS: Numeric Rating Scale
RCT: randomized controlled trial
AP group: the acupuncture group
SAP group: the sham acupuncture group
NSAID: non-steroidal anti-inflammatory drug
COX: cyclooxygenases
TRUS: transrectal ultrasonography
EMRI: enhanced magnetic resonance imaging
SAE: serious adverse events
ASCRS: American Society of Colon & Rectal Surgeons
CRF: case report form
CCF-IS: Cleveland Clinic Florida Incontinence Score
GIS: Garg Incontinence Scores
PSQI: Pittsburgh Sleep Quality Index
SDS: Self-rating Depression Scale
SAS: Self-Rating Anxiety Scale
QoR-15: Quality of Recovery Scale
TRUS: Transrectal Ultrasound
EMRI: Endorectal Magnetic Resonance Imaging
EDC: electronic data capture
DSMC: data and safety monitoring committee
AIC: Akaike Information Criterion
