# Correction: The efficacy of acupuncture in relieving postoperative pain in patients with low simple anal fistula: Protocol of a prospective, randomised, controlled trial

**DOI:** 10.1371/journal.pone.0321696

**Published:** 2025-04-01

**Authors:** Min Yang, De Zheng, Xingtao Jin, Huili Tang, Weiwei Cao, Qianqian Ye, Yin Qu, Zubing Mei

There is an error in Fig 3. The labeling is incorrect. Please see the correct Fig 3 here.

**Fig 3:**
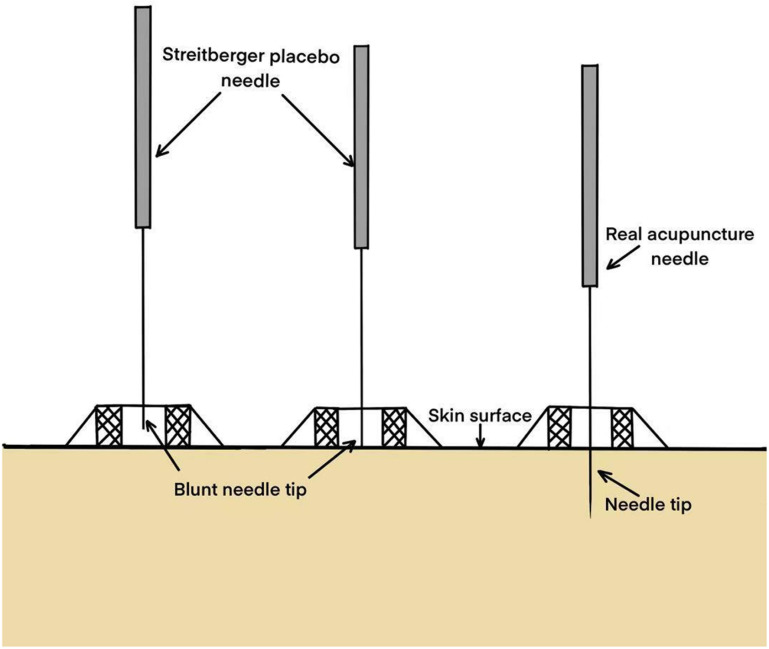
Streitberger placebo needle.
