# Supplementary material for: The efficacy of acupuncture in relieving postoperative pain in patients with low simple anal fistula: Protocol of a prospective, randomised, controlled trial
Source: PLoS One. 2025 Jan 24;20(1):e0317902. doi: 10.1371/journal.pone.0317902 (PMC11761113; doi:10.1371/journal.pone.0317902)
Supplement: S2 File — (PDF) [file pone.0317902.s002.pdf]

Evaluating the efficacy of Acupuncture in relieving postoperative pain of Low Simple Anal Fistula: Protocol of a Prospective, Randomized, Controlled Trial

Principle Investigator: Prof. De Zheng, professor of the Department of Anorectal Surgery, Shuguang Hospital, Shanghai University of Traditional Chinese Medicine.

E-mail: zd1232@sina.com

Address:528 Zhangheng Road, Shanghai 201203, China

Version: 2.0

Date: May 15, 2024

Abstract

Introduction: Anal fistula is a chronic inflammatory condition characterized by the formation of an abnormal channel connecting the anal canal, rectum, and perianal skin. This condition often arises due to inadequate drainage of abscesses, resulting in recurrent infections and significant morbidity. Despite the availability of various treatment modalities, postoperative pain remains a significant challenge, significantly impacting the recovery process and overall quality of life for affected individuals. The present study aims to rigorously evaluate the effectiveness of acupuncture in mitigating postoperative pain following surgical intervention for anal fistula.

Methods and analysis: This single-center, patient-blinded, assessor-blinded, placebo-controlled randomized controlled trial (RCT) will be conducted at a tertiary referral hospital. A total of 66 patients with low simple anal fistula will be randomized in a 1:1 ratio to receive either acupuncture or sham acupuncture. The primary outcome is the difference in the Numeric Rating Scale (NRS) pain scores before and after acupuncture 6 hours post-surgery. Secondary outcomes include postoperative analgesic usage, urinary retention incidence, sleep quality, psychological state, and overall recovery quality. Data will be analyzed using repeated measures ANOVA and chi-square tests.

Discussion: This study aims to evaluate acupuncture as an adjunctive therapy for postoperative pain relief of anal fistula surgery. It is the first RCT combining acupuncture with standard analgesic interventions in this context, addressing a critical gap in medical literature. The trial's strengths include a comprehensive outcome assessment and a robust design ensuring internal validity. Limitations include its single-center design, small sample size, and short follow-up

period, which may affect generalizability and long-term applicability. Future studies should expand the sample size, explore additional acupoints, and involve multi-center trials to validate these findings. This well-designed trial will provide valuable evidence for acupuncture's role in postoperative pain management and its potential to enhance patient recovery.

Study Protocol

1.Study background

Anal fistula is a chronic inflammatory condition characterized by the development of an aberrant epithelialized tract connecting the anal canal, rectum, and the perineal skin. This condition often arises as a consequence of inadequate drainage of abscesses within the anal and rectal region, giving rise to recurrent episodes of infection. Additionally, anal fistulas may be caused by rectal or anal canal malignancies, Crohn's disease, ulcerative colitis, and various other pathologies. Other contributory factors may include fecal retention, irritation from foreign bodies, and traumatic infections. Clinically, patients may present with symptoms such as severe discomfort, secretion discharge, fecal incontinence, and leakage[1-3]. The incidence rate of anal fistula has been rising year by year, from 1-2 cases per 100,000 people reported in the 1980s to over 20 cases per 100,000 people today. The disease is more common in young and middle-aged males aged 20-40. In China, the incidence rate of anal fistula is about 1.7%-3.6%, with a peak occurrence in young and middle-aged individuals, and the number of male patients is 2-6 times that of female patients[4]. Anal fistulas exhibit a recalcitrant nature in spontaneous healing, necessitating prompt intervention to mitigate the risk of recurrence. The therapeutic approach is primarily categorized into pharmacological therapy and surgical interventions. During the acute phase, pharmacological management can assist in mitigating swelling and discomfort, yet its curative potential is limited, predisposing patients to potential recurrences. Consequently, surgical management is often preferred in clinical practice for the

treatment of anal fistulas. Surgical options include incision and drainage, thread draining, fistulotomy, fistulectomy, and fistula tract filling, with the selection of the appropriate surgical technique being dependent on the specific type of fistula. It is noteworthy that postoperative complications associated with anal fistula surgery may encompass pain, bleeding, constipation, and urinary retention. A case study by Nikhil C and others found that for patients under 50, pain was the primary postoperative complication, significantly higher than other common complications[5]. Therefore, effectively preventing and treating postoperative pain from anal fistulas is an urgent issue that colorectal surgeons need to address.

From a physiological perspective, the discomfort experienced by individuals as surgical instruments are utilized during anal fistula surgery, resulting in interactions with the skin and muscles, exhibits notable parallels with somatic and visceral pain mechanisms. During the surgical treatment of an anal fistula, the human body responds by activating inflammatory cells in proximity to the perianal injury site. This activation triggers the generation of numerous inflammatory mediators, including bradykinin, serotonin, and prostaglandins. These mediators contribute to the perception of intense pain, stemming from the persistent contraction of the sphincter muscle[6]. Decomposition products of the extracellular matrix and adenosine triphosphate released by necrotic cells activate the receptors on the surface of hypertrophic cells, leading to degranulation and an increased release of inflammatory mediators. This process is fundamental in the pathophysiology of various inflammatory diseases and plays a crucial role in the immune response[7]. The

specific mechanism by which inflammatory factors affect postoperative pain involves stimulating nociceptors, which transmit electrical impulses to the central nervous system. Their continuous activation increases the excitability of the receptors, thus adjusting the pain threshold. Inflammatory factors increase the sensitivity of excitatory receptors, and excitatory neuropeptides such as substance P and glutamate interact with membrane receptors coupled to G-proteins in neurons, lowering the action potential threshold of dorsal horn secondary neurons. This creates a "sensitization" phenomenon from normal subthreshold stimuli, leading to pain[8-10]. Given the pre-existing association of anal fistulae with pain resulting from local inflammatory responses, this phenomenon leads to heightened sensitivity of surrounding nociceptors and subsequent release of diverse inflammatory factors, thereby exacerbating postoperative pain perception[11].

Post-anal fistula surgery pain is unique due to the anatomy of the perianal region. Below the dentate line, it is controlled by the autonomic nervous system, and the peripheral nerves are abundant. During defecation after anal fistula excision surgery, the contraction of the sphincter muscle is required, which stimulates the nerves and causes pain. After defecation, if fecal residue remains on the surgical wound in the anal canal, bacteria in the feces can also stimulate the wound nerves. This leads to local tissue vasodilation and increased capillary wall permeability in the anal region. In this situation, plasma and blood components such as neutrophils are secreted through the vessel walls into the surrounding area of the anal wound[12]. Additionally, the secretion of polypeptide substances such as bradykinin increases,

altering vascular permeability, causing spasms of the anal smooth muscle, and inducing vasodilation. Furthermore, the increased secretion of proteinases such as blood fibrinolytic enzymes and kinin-releasing enzymes converts kininogen into kinin, thereby altering vascular permeability and exacerbating inflammatory edema, leading to pain. Apart from these factors, psychological stress and surgical techniques can also affect postoperative pain.

Modern medical treatment for post-anal fistula surgery pain employs various drugs and methods, including non-steroidal drugs, opioid drugs, calcium channel blockers, injectable drugs, and other adjunctive medications. Non-steroidal drugs such as aspirin and indomethacin reduce inflammation and pain by inhibiting prostaglandin synthesis, but may have adverse effects on kidney function[13]. Opioid drugs such as morphine and codeine have analgesic effects on the central nervous system, but can lead to dependency and constipation[14, 15]. Calcium channel blockers like diltiazem reduce pain by lowering anal resting pressure[16]. Injectable drugs such as compound menthol injection and compound methylene blue injection alleviate pain by directly acting on nerves or muscles[17]. Other drugs like gabapentin and diazepam are also used for pain management, each with its own side effects and limitations, and their use for analgesia in clinical practice is currently limited[18, 19].

Acupuncture has been widely used to alleviate pain, and its analgesic effects have been reported worldwide. Several studies have shown that acupuncture can relieve postoperative pain, such as in cardiac surgery[20], laparoscopic cholecystectomy[21], and hemorrhoidectomy[22]. Therefore, it is reasonable to

speculate that acupuncture may also alleviate post-anal fistula surgery pain. Despite the positive results from these previously published studies, the reliability of the findings is compromised due to small sample sizes, inadequate randomization, and lack of blinding. Therefore, high-quality evidence is needed to confirm previous findings and develop a protocol for using acupuncture to treat postoperative pain.

Given the aforementioned reasons, we have designed a randomized controlled trial (RCT) that incorporates appropriate randomization methods, strict allocation concealment measures, and blinding techniques to meticulously evaluate the effectiveness of acupuncture in mitigating post-anal fistula surgery pain. Our primary objectives are twofold: firstly, to ascertain whether acupuncture serves as a viable adjuvant in alleviating postoperative pain; secondly, to assess its significance in facilitating the recovery process following anal fistula surgery, encompassing improvements in postoperative sleep quality, enhancement of patient psychological well-being, and reduction in analgesic usage. The ultimate goal of this study is to furnish high-quality evidence that can contribute to the standardization of acupuncture treatment protocols for managing post-anal fistula surgery pain. By exploring acupuncture as a complementary therapy in the prevention and management of post-anal fistula surgery pain, we aim to identify safer and more efficacious treatment strategies, thereby reducing postoperative pain and analgesic consumption among patients undergoing anal fistula surgery, and ultimately enhancing their overall quality of life.

2.Study object

Patients eligible for inclusion should meet the following criteria: diagnostic criteria consistent with low simple anal fistula, distance from the external opening of the anal fistula to the anal verge ≤3cm, age between 18 and 65 years, no absolute contraindication to anal fistulotomy, voluntary participation in the trial, ability to fully cooperate with treatment, and signed informed consent form. Exclusion criteria includes those who did not meet the inclusion criteria, patients with hemorrhoids, anal fissure, perianal abscess, anorectal tumor, Crohn's disease, perianal skin disease, and other perianal diseases, female patients currently in pregnancy and lactation or special physiological periods, patients with severe hypertension, diabetes, coronary heart disease or other chronic medical diseases, malignant tumors or coagulation disorders and other surgical contraindications, patients with severe mental illness or cognitive impairment with communication difficulties, patients with contraindications to acupuncture such as ulcer, infection, or scar on the skin of the intended acupuncture site, and those who had received acupuncture treatment in the past 3 months.

3.Study design

This is a single-center, patient-blinded and assessor-blinded, placebo-controlled randomized controlled trial (RCT) that will be conducted at a tertiary referral hospital. Eligible patients will be randomly assigned in a 1:1 ratio to either the acupuncture group or the sham acupuncture group. Prior to enrollment in the trial, all patients will be required to provide written informed consent.

4.Sample size estimation

In November 2023, we have conducted a meticulously designed pilot study to

examine potential differences in NRS scores between patients receiving acupuncture and those receiving sham acupuncture. The determination of the sample size was meticulously conducted, relying on a thorough analysis of data garnered from a preliminary study utilizing the PASS 2021 software (NCSS, Kaysville, Utah, USA). Notably, during the pilot study, a clinically significant treatment effect was observed, with a distinct difference of 0.8 points in NRS scores between the two groups, measured before and six hours after the acupuncture intervention.

To ensure the accuracy and reliability of our findings, a two-sided test was employed in the calculation of the sample size for comparing means within a completely randomized design. The parameters used in this calculation included $\alpha=0.05$, $\beta=0.10$, $\mu 1=1.6$, $\mu 2=2.4$, and $\sigma=0.91$. By inputting these parameters into the PASS software, a minimum required sample size of N=29 was determined. However, to account for potential dropouts, a 10% dropout rate was factored into the final calculation, resulting in a revised sample size of N=33, with 66 cases evenly distributed across both groups.

To ensure the randomization process is both fair and transparent, the sealed envelope method will be utilized. Each envelope will contain a slip of paper clearly labeled as either "real acupuncture" or "sham acupuncture." Once a patient is enrolled in the study, they will be randomly assigned to an envelope, with their name recorded on the envelope for identification purposes. The acupuncture doctor responsible for the intervention will open the envelope, perform the acupuncture accordingly, and then reseal the envelope. During the entire study period, the envelope will remain in

the possession of the acupuncturist and will be reopened prior to each acupuncture session to verify the group assignment. This ensures that the randomization process remains concealed from patients and other study personnel, with only the acupuncturist aware of the specific group assignment. Upon completion of the study, the envelopes will be opened to retrieve the group assignment data. This rigorous and methodical approach ensures the integrity of the randomization process and maintains the highest standards of scientific rigor throughout the study.

5.Interventions

(1) Surgical Method

Both cohorts of patients will undergo anal fistula excision under intravenous anesthesia, administered by a highly skilled and experienced senior physician, to minimize postoperative variations. Patients will be positioned laterally, and the surgical site will undergo routine disinfection and anesthetization. Upon satisfactory anesthesia, a probe will be inserted through the external orifice of the fistula, traversing the fistula tract, and emerging through the internal orifice. The perianal skin and subcutaneous tissue along the probe's path will be excised to ensure complete removal of the fistula tract. The surgical wound will be left open to facilitate drainage, and upon completion of the surgical procedure, external dressings will be applied to the wound, followed by pressure bandaging.

(2) Postoperative Pain Management Interventions

1.Pain medication

If patients report pain with an NRS (Numerical Rating Scale) score ranging from 4 to 7, they will be prescribed 60mg of Loxoprofen Sodium for oral administration. Conversely, in cases where the NRS score exceeds 7, patients will be administered an intramuscular injection containing 30mg of Ketorolac Tromethamine. Loxoprofen Sodium, a non-steroidal anti-inflammatory drug (NSAID), functions by suppressing the activity of cyclooxygenase within the body, leading to a reduction in the production of inflammatory mediators, thereby effectively alleviating symptoms of pain, inflammation, and fever[23].

2.Standard acupuncture needles

Acupuncture needles is manufactured by Suzhou Medical Supplies Factory Co., Ltd., "Hua Tuo Brand" filiform needles (size: 0.25mm × 40mm).

3.Placebo acupuncture devices

Streitberger placebo needles (Streitberger placebo device [Asia-med GmbH]). This device looks like a real acupuncture needle but has a blunter tip, and the needle within the handle is not fixed. When the tip touches the skin, the needle retracts into the handle, and it is then fixed with a plastic ring covered with a plastic piece, creating not only a sensation of pricking but also the illusion that the needle has actually penetrated the skin. This placebo acupuncture device has been proven to be a credible placebo control in acupuncture trials among the Chinese population with experience in acupuncture and is widely used in acupuncture research[24-26]

4.Acupoint selection

① Changqiang point: the midpoint of the line between the tip of the coccyx and

the anus;

② Chengshan point (bilateral): in the middle of the back of the leg, between Weizhong point and Kunlun point, when the leg or foot is stretched, the sharp Angle depression occurs under the gastrocnemius muscle belly;

③ Upper Juxu point (bilateral): in the anterolateral side of the calf, when the calf is 6 cun below the nose, a transverse finger (middle finger) from the anterior edge of the tibia.

④ Erbai point (bilateral) : the anterior area of the forearm, 4 cun on the transverse line of the distal side of the palmar wrist, on both sides of the flexor carpi radialis tendon.

⑤ Hegu point (bilateral) : on the back of the hand, between the first and second metacarpal bones, when the midpoint of the radial side of the second metacarpal bone.

All patients will receive acupuncture for 30 minutes each for a total of 7 days. On the operation day, acupuncture will be given 6 hours after the operation, and then acupuncture will be given once every morning and evening.

The experimental group will be treated with acupoint acupuncture. The patient will be placed in a lateral decubitus position on the edge of the bed with the knee bent, exposing the lumbosacral region and limbs. Local disinfection of the acupoint area will be performed using 75% alcohol. A sterile acupuncture needle of 0.25mm × 40mm will be selected to stimulate the above acupoints. Straight needling will be applied at Shangjuxu (ST 37) and Erbai (ST 2), with a depth of 0.5-1 cun. Oblique needling will be applied at Changqiang point with a depth of 0.5-1 cun. The acupoints

of Chengshan and Hegu will be needled straight, with a depth of 1 to 2 cun. According to the patient's feeling of acid, numbness, and distension, the standard of gas intake will be defined. The needle will be retained for 30 minutes by lifting and thrusting and twirling. After the needle is removed, the needle hole will be rapidly pressed with a cotton swab until there is no bleeding. The same procedure will be performed at the same time every day.

The control group will be treated with sham acupuncture. After routine position and disinfection, the patients will be treated with Streitberger's sham acupuncture device. The rest will be the same as the experimental group.

6.Clinical observation index

(1) Baseline data statistics

The patient's age, gender, BMI, occupation, education background (primary school and below, junior or senior high school, university and above), smoking, drinking, expectation of acupuncture analgesic effect (completely believe, believe, doubt, completely oppose), preoperative NRS pain score, sleep quality score, SAS score and SDS score will be collected and evaluated as baseline.

(2) Primary outcome measure

The primary outcome of this study is the difference of NRS score before (H6a) and after (H6b) acupuncture 6h after surgery. Compared to other measurement methods such as the Visual Analogue Scale, numbers are easier for patients to grade the intensity of pain. As a patient-selected quantification result of pain, the Numeric Rating Scale (NRS) is highly feasible in clinical research and practice. Patients can

choose the most appropriate number to describe their pain, and it has been proven to be reliable, effective, and sensitive. It is an 11-point scale ranging from 0 to 10, where 0 represents no pain and 10 represents the most severe pain imaginable.

(3) Secondary outcome measure

Secondary outcomes in this study include post-operative NRS pain scores on days 1, 3, and 7, and post-operative analgesic medication usage. The usage of analgesic medication, including oral Loxoprofen Sodium or temporary use of Ketorolac Tromethamine Injection for muscular injection, will be recorded up to the seventh day post-operation, and the usage rate of post-operative analgesics will be calculated by dividing the number of people who use additional pain medication from the first day post-operation by the total number of people in each group. The incidence of post-operative urinary retention will be assessed using a points system where 0 points indicate smooth urination without difficulties, 2 points suggest slight difficulty but no treatment needed, 4 points indicate difficulty urinating with successful conservative treatment, and 6 points denote significant difficulty requiring catheterization. Post-operative sleep quality will be observed and recorded using the Pittsburgh Sleep Quality Index (PSQI) from the day before the operation to the third day post-operation, with the PSQI score ranging from 0 to 21 points. The psychological state of patients will be evaluated using the Self-rating Depression Scale (SDS) and the Self-Rating Anxiety Scale (SAS) one day before and on days 1 and 7 post-operation, where higher scores indicate higher levels of anxiety and depression. Post-operative recovery quality will be assessed using the 15-item Quality of Recovery Scale (QoR-15) on days 1, 3, and 7 post-operation, covering five dimensions: patient comfort, receiving help, emotional state, physical independence, and pain, with each item scored from 0 to 10. After completing the acupuncture treatment on the third day post-operation, the success of blinding will be tested by asking patients which type of treatment they believe they received: acupuncture group, sham acupuncture group, or uncertain. Additionally, upon discharge, patients will

be asked whether they would be willing to receive acupuncture for pain control in the future and if they would recommend acupuncture for pain control to others.

References

1. Amato A, Bottini C, De Nardi P, Giamundo P, Lauretta A, Realis Luc A, Piloni V. Evaluation and management of perianal abscess and anal fistula: SICCR position statement. Tech Coloproctol. 2020;24(2):127-43. Epub 20200123. doi: 10.1007/s10151-019-02144-1. PubMed PMID: 31974827.
2. Pescatori M. Surgery for anal fistulae: state of the art. Int J Colorectal Dis. 2021;36(10):2071-9. Epub 20210531. doi: 10.1007/s00384-021-03917-7. PubMed PMID: 34057576.
3. Wasmann KA, de Groof EJ, Stellingwerf ME, D'Haens GR, Ponsioen CY, Gecse KB, et al. Treatment of Perianal Fistulas in Crohn's Disease, Seton Versus Anti-TNF Versus Surgical Closure Following Anti-TNF [PISA]: A Randomised Controlled Trial. J Crohns Colitis. 2020;14(8):1049-56. doi: 10.1093/ecco-jcc/jjaa004. PubMed PMID: 31919501; PubMed Central PMCID: PMCPMC7476637.
4. Zeng XD, Zhang Y. Surgical treatment of anal fistula. Chinese Journal of Gastrointestinal Surgery. 2014;17(12).
5. Crain N, Aboulian A. Unplanned Returns to Care within Seven Days after Anorectal Surgery: Can they be Avoided? Am Surg. 2019;85(1):92-7. PubMed PMID: 30760352.
6. Nakanishi R, Konishi T, Nakaya E, Zaitsu Y, Mukai T, Yamaguchi T, et al. Predisposing factors and clinical impact of high-output syndrome after sphincter-preserving surgery with covering ileostomy for rectal cancer: a retrospective single-center cohort study. Int J Clin Oncol. 2021;26(1):118-25. Epub 20200909. doi: 10.1007/s10147-020-01781-z. PubMed PMID: 32902781.
7. Lan L, Huang YG, Shen L. Research progress of inflammatory response and immune regulation mechanism related to postoperative chronic pain. Chinese Journal of Pain Medicine. 2017;23(3):161-4. doi: 10.3969/j.issn.1006-9852.2017.03.001.
8. Voscopoulos, C., Lema, M. When does acute pain become chronic? BJA: The British Journal of Anaesthesia. 2010.
9. Woolf CJ, Thompson SWN. The induction and maintenance of central sensitization is dependent on N-methyl-D-aspartic acid receptor activation; implications for the treatment of post-injury pain hypersensitivity states. Pain. 1991;44(3):293-9. doi: 10.1016/0304-3959(91)90100-c. PubMed PMID: 1828878.
10. Butterworth JF. Morgan & Mikhail's clinical anesthesiology. Mcgraw Hill. 2013.
11. Wang Y, Liu Z, Chen S, Ye X, Xie W, Hu C, et al. Pre-surgery beliefs about pain and surgery as predictors of acute and chronic post-surgical pain: A prospective cohort study. Int J Surg. 2018;52:50-5. Epub 20180217. doi: 10.1016/j.ijsu.2018.02.032. PubMed PMID: 29462739.
12. Claesson-Welsh L. Vascular permeability--the essentials. Ups J Med Sci. 2015;120(3):135-43. Epub 20150729. doi: 10.3109/03009734.2015.1064501. PubMed PMID: 26220421; PubMed Central PMCID: PMCPMC4526869.
13. Gottlieb S. COX 2 inhibitors may increase risk of heart attack. Bmj. 2001;323(7311):471. PubMed PMID: 11532833; PubMed Central PMCID: PMCPMC1121075.
14. Elton RJ, Chaudhari S. Sepsis in obstetrics. Bja Education. 2015;(5):259-64.
15. Wang Y, Jang K. Progress in pathological mechanism and therapy of opioid-induced constipation **Chinese Journal of Clinical Oncology**. 2021;48(16):852-7.

16. Ibrahim S, Natarajan R, Mohamed ZAR, Loganathan M. A COMPARATIVE STUDY OF TOPICAL 2% DILTIAZEM WITH LATERAL SPHINCTEROTOMY IN THE TREATMENT OF CHRONIC FISSURE IN-ANO. Annals of Tropical Medicine and Public Health. 2020;23(15).
17. Zheng XB, Yang WZ, Duan HY, Yang WP. Clinical study of compound menthol injection in the treatment of postoperative anal pain. Clinical Medicine of China. 2002;(05):79-80.
18. Nakhli MS, Kahloul M, Jebali C, Frigui W, Naija W. Effects of Gabapentinoids Premedication on Shoulder Pain and Rehabilitation Quality after Laparoscopic Cholecystectomy: Pregabalin versus Gabapentin. Pain Res Manag. 2018;2018:9834059. Epub 20180709. doi: 10.1155/2018/9834059. PubMed PMID: 30123399; PubMed Central PMCID: PMCPMC6079331.
19. Liu XP, Zang YP, Yan CX, Bai JH. Observation on the analgesic effect of intravenous low dose droperidol and diazepam in forceps curettage. Maternal and Child Health Care of China. 2011;26(15):2369-70.
20. Roshanzamir S, Haririan Y, Ghaderpanah R, Jahromi LSM, Dabbaghmanesh A. Investigation of the Effects of Acupuncture on Post-Operative Chest Pain after Open Heart Surgery. J Acupunct Meridian Stud. 2023;16(4):133-8. doi: 10.51507/j.jams.2023.16.4.133. PubMed PMID: 37609768.
21. Li MA. Study on the effect of acupuncture on reducing pain after laparoscopic cholecystectomy [Master's thesis]: **Guangzhou University of Chinese Medicine**; 2021.
22. Zheng HL, Zhou XC, Yu L, Lu BY. Effect of Liangxue Dihuang Decoction combined with acupuncture on postoperative wound recovery and pain in patients with mixed hemorrhoids. Journal of Sichuan Traditional Chinese Medicine. 2023;(009):041.
23. Zhao D, Chen Z, Hu S, Lin J, Shao Z, Wang G, et al. Efficacy and Safety of Loxoprofen Hydrogel Transdermal Patch Versus Loxoprofen Tablet in Chinese Patients with Myalgia: A Double-Blind, Double-Dummy, Parallel-Group, Randomized, Controlled, Non-Inferiority Trial. Clin Drug Investig. 2019;39(4):369-77. doi: 10.1007/s40261-019-00756-x. PubMed PMID: 30725315; PubMed Central PMCID: PMCPMC6443607.
24. Xie CC, Wen XY, Jiang L, Xie MJ, Fu WB. Validity of the "streitberger" needle in a chinese population with acupuncture: a randomized, single-blinded, and crossover pilot study. Evid Based Complement Alternat Med. 2013;2013:251603. Epub 20130801. doi: 10.1155/2013/251603. PubMed PMID: 23983775; PubMed Central PMCID: PMCPMC3747414.
25. Fu C, Zhao N, Liu Z, Yuan LH, Xie C, Yang WJ, et al. Acupuncture Improves Peri-menopausal Insomnia: A Randomized Controlled Trial. Sleep. 2017;40(11). doi: 10.1093/sleep/zsx153. PubMed PMID: 29029258.
26. Schneider A, Enck P, Streitberger K, Weiland C, Bagheri S, Witte S, et al. Acupuncture treatment in irritable bowel syndrome. Gut. 2006;55(5):649-54. Epub 20050908. doi: 10.1136/gut.2005.074518. PubMed PMID: 16150852; PubMed Central PMCID: PMCPMC1856122.
