# Supplementary material for: The efficacy of acupuncture in relieving postoperative pain in patients with low simple anal fistula: Protocol of a prospective, randomised, controlled trial
Source: PLoS One. 2025 Jan 24;20(1):e0317902. doi: 10.1371/journal.pone.0317902 (PMC11761113; doi:10.1371/journal.pone.0317902)
Supplement: S3 File — (DOCX) [file pone.0317902.s003.docx]

*Statistical Analysis Plan*

**Title:** Evaluating the efficacy of acupuncture in relieving postoperative pain of low simple anal fistula

**Principle Investigator:** Prof. De Zheng, professor of the Department of Anorectal Surgery, Shuguang Hospital, Shanghai University of Traditional Chinese Medicine.

**E-mail:** zd1232@sina.com

Address:528 Zhangheng Road, Shanghai 201203, China

**Version:** 1.0

**Date:** June 1, 2024

1. **Data Handling**

**1.1 Data collection and management methods**

To ensure accurate and efficient data management, we will implement an electronic data capture (EDC) system. The introduction of this system will not only help improve data management efficiency, but also ensure data quality and security during the research process. To ensure the efficient operation of the EDC system, we will employ a dedicated clinical research assistant who will be responsible for comprehensive supervision of data management and monitoring of research progress. This individual will possess extensive experience in clinical research and demonstrate meticulous skills in data management to effectively control both data quality and research progress during the study.

The clinical research assistant will securely access the EDC system through a stringent personal electronic account, which will be safeguarded by robust confidentiality measures to ensure exclusive access and modification rights for authorized personnel. This approach guarantees the utmost security of collected patient information throughout the input and transmission process to prevent any potential data leakage or unauthorized access.

To protect the privacy and rights of patients, all data participating in the study will be anonymized under the supervision of the clinical research assistant. This measure is intended to eliminate the direct association between personal information and data, thereby ensuring that the privacy of patients is fully protected. At the same time, the clinical research assistant will also be responsible for the detailed entry, comprehensive inspection and comprehensive management of data to ensure the accuracy and completeness of the data.

**1.2 Handling of missing data**

We will conduct a thorough descriptive analysis to understand the pattern and extent of missing data. This will include reporting the percentage of missing data for each variable and examining whether the missingness is related to other variables. For simple cases of missing data, we will use methods such as mean imputation or regression imputation to estimate missing values based on available data. For more complex cases, multiple imputation will be used to create several complete datasets by imputing missing values multiple times, reflecting the uncertainty of the missing data. These datasets will be analyzed separately, and the results will be combined to produce final estimates. We will also perform sensitivity analyses to assess how different methods of handling missing data impact the study results. This helps in understanding the robustness of the findings under various assumptions about the missing data mechanism.

During the study, continuous monitoring of data collection processes will be implemented to minimize the occurrence of missing data. Any patterns or systematic issues identified will be addressed promptly. All instances of missing data and the methods used to handle them will be meticulously documented. This documentation will include the reasons for missing data, the assumptions made, and the specific imputation or analysis techniques applied. The extent of missing data, the methods used to handle it, and the results of sensitivity analyses will be transparently reported in the study's results section. This will include a discussion on how missing data might have affected the study's findings and the potential limitations introduced by the handling methods.

1. **Statistical Methods**

**2.1 Statistical Software**

During the study process, all data will be observed and recorded by the same individual in accordance with standardized protocols. The research data will be entered and organized by an independent third party not involved in this clinical trial. SPSS software (IBM SPSS 25.0, SPSS Inc) will be used for statistical analysis of the collected data. The normality of measurement data is verified by Shapiro-Wilk test, and the homogeneity of variance is assessed using Levene's test. All statistical analyses will employ two-sided tests, with P-values less than 0.05 considered statistically significant.

**2.2 Primary and secondary outcome analysis**

The primary outcome, the difference of NRS score between H6a and H6b after surgery, along with secondary outcome scores for CCF-IS & GIS, PSQI, SAS & SDS and QoR-15, are continuous data. If they follow a normal distribution with equal variances, they will be described using the mean and standard deviation. Otherwise, they will be described using the median and interquartile range. The incidence of complications and the usage rate of postoperative analgesics are categorical data, will be analyzed using the Chi-square test. Efficacy indicators that require repeated measurements, such as NRS, CCF-IS & GIS scores, will be analyzed using repeated measures ANOVA.

**2.3 Analysis populations**

（1）Intent-to-Treat (ITT) Population

The ITT analysis aims to provide an unbiased estimate of the treatment effect by reflecting real-world scenarios, including non-adherence and dropouts. It helps to understand the effectiveness of the intervention under routine clinical conditions. All participants who were randomized to either the acupuncture group or the sham acupuncture group will be included in the ITT population. Participants will be analyzed in the groups to which they were originally assigned, regardless of protocol deviations, non-compliance, or withdrawal. Missing data in the ITT population will be handled using appropriate imputation methods as described in the "Handling of Missing Data" section to ensure that all randomized participants are included in the analysis.

（2）Per-Protocol (PP) Population

The PP analysis aims to evaluate the efficacy of the treatment when administered precisely as intended. It helps to determine the potential benefits of the intervention in a controlled environment where adherence is maximized. Participants who fully adhered to the study protocol, including attendance at all scheduled acupuncture sessions and compliance with the treatment regimen, will be included in the PP population. Significant protocol deviations that will lead to exclusion from the PP population include major violations such as incorrect group assignment, failure to receive the allocated intervention, or incomplete follow-up.

Criteria for PP population Inclusion:

*Participants must have received the full course of the allocated treatment. *Participants must have completed all primary and secondary outcome assessments.

*Any deviations from the protocol that do not significantly impact the study's integrity may still be included based on predefined criteria.

（3）As-Treated Population:

Participants will be analyzed according to the treatment they actually received, regardless of their initial randomization. This analysis helps to understand the effects of the intervention based on actual treatment exposure.

（4）Safety Population:

All participants who received at least one session of the treatment (either acupuncture or sham acupuncture) will be included in the safety population. This analysis focuses on evaluating the safety and adverse effects of the interventions.

1. **Baseline Comparability**

The patient's age, gender, BMI, occupation, education background, smoking, drinking, disease duration, antibiotic drug use, previous perianal surgery, expectation of acupuncture analgesic effect, preoperative NRS pain score, sleep quality score, SAS score and SDS score will be collected and evaluated as baseline.

Baseline analysis includes categorical variables such as gender, occupation, educational background, smoking habit, drinking habit, disease duration, antibiotic drug use, previous perianal surgery and expectations for the analgesic effect of acupuncture, which will be expressed as patient counts and respective percentages, with intergroup comparisons performed using the Chi-square test. Continuous variables such as age, BMI, preoperative NRS pain scores, sleep quality scores, preoperative SAS scores, and SDS scores will be analyzed based on their distribution. If the data are normally distributed with homogeneity of variance, they will be reported as mean ± standard deviation and compared using the independent samples t-test; otherwise, they will be presented as medians and analyzed using the Mann-Whitney U test.

1. **Interim Analysis and Stopping Rules**

An interim analysis will be conducted when 50% of the participants have completed the primary outcome assessment (6 hours post-surgery pain scores). Additional interim analyses may be conducted as necessary, depending on emerging safety data or unexpected efficacy signals. The interim analysis will be conducted in a blinded manner to avoid introducing bias. The DSMC will have access to unblinded data, but investigators and participants will remain blinded to treatment assignments unless safety concerns necessitate unblinding.

The trial may be stopped early for safety if there is evidence of significant harm to participants in any treatment arm. The DSMC will monitor adverse events and serious adverse events (SAE) closely, with predefined thresholds for halting the trial.

Interim analysis will employ appropriate statistical methods to compare treatment groups. Adjustments for multiple looks at the data will be made using methods such as the O’Brien-Fleming or Lan-DeMets alpha spending functions to control the overall type I error rate.

The DSMC will provide a detailed report of the interim analysis findings and recommendations. Based on the DSMC’s recommendations, the trial steering committee will make final decisions regarding the continuation, modification, or termination of the trial. Results of the interim analysis will be communicated to relevant stakeholders, including the trial sponsor and ethics committee. Any decisions to stop or modify the trial will be documented and communicated to all investigators and participants.
